# 

**Published:** 1894-06

**Authors:** 


### CONTENTS.

COMMUNICATIONS.

Diseases of the Maxillary Bones and their Periosteum........................ 361
The Indications of Dental Exostosis........................................ 269
Diarrhœa of Dentition...................................................... 272
Articulation of Artificial Teeth............................................ 273

SELECTIONS.

Longevity .................................................................. 280
Synthetical Chemistry and its Relation to the Materia Medica of the Future.................................................................. 293
New York Academy of Medicine................................................ 297
Increasing Longevity........................................................ 299
Sir Andrew Clark—A Reminiscence.............................................. 300
Improved Cleft Palate Operation.............................................. 302
Can Women Farm?............................................................. 304
The Influence of Odors upon the Voice........................................ 304
The Prevention of Disease.................................................... 305
The Price of Glass Eyes in England........................................... 306

NOTICES.

The Kentucky State Dental Association......................................... 307
Illinois State Dental Society................................................. 308
The Indiana State Dental Society.............................................. 308

EDITORIAL.

A Change in the Dental Law of Ohio............................................ 309
The American Dental Association.............................................. 310
Bibliography................................................................. 311
Obituary..................................................................... 312

